# C/EBPβ-1 promotes transformation and chemoresistance in Ewing sarcoma cells

**DOI:** 10.18632/oncotarget.14847

**Published:** 2017-01-27

**Authors:** Jamie D. Gardiner, Lisa M. Abegglen, Xiaomeng Huang, Bryce E. Carter, Elizabeth A. Schackmann, Marcus Stucki, Christian N. Paxton, R. Lor Randall, James F. Amatruda, Angelica R. Putnam, Heinrich Kovar, Stephen L. Lessnick, Joshua D. Schiffman

**Affiliations:** ^1^ Department of Pediatrics, Huntsman Cancer Institute, University of Utah, Salt Lake City, UT, USA; ^2^ School of Medicine, University of Utah, Salt Lake City, UT, USA; ^3^ ARUP Institute for Clinical and Experimental Pathology^®^, Salt Lake City, UT, USA; ^4^ Department of Orthopaedic Surgery, Sarcoma Services, University of Utah, Salt Lake City, UT, USA; ^5^ Department of Pediatrics, Internal Medicine and Molecular Biology, University of Texas Southwestern, Dallas, TX, USA; ^6^ Department of Pathology, University of Utah, Salt Lake City, UT, USA; ^7^ Children's Cancer Research Institute, St. Anna Kinderkrebsforschung, Vienna, Austria; ^8^ Center for Childhood Cancer and Blood Disorders, Nationwide Children's Hospital, and the Division of Pediatric Heme/Onc/BMT, The Ohio State University, Columbus, OH, USA

**Keywords:** Ewing sarcoma, C/EBPβ, ALDH1A1, chemoresistance, biomarker

## Abstract

*CEBPB* copy number gain in Ewing sarcoma was previously shown to be associated with worse clinical outcome compared to tumors with normal *CEBPB* copy number, although the mechanism was not characterized. We employed gene knockdown and rescue assays to explore the consequences of altered *CEBPB* gene expression in Ewing sarcoma cell lines. Knockdown of EWS-FLI1 expression led to a decrease in expression of all three C/EBPβ isoforms while re-expression of EWS-FLI1 rescued C/EBPβ expression. Overexpression of C/EBPβ-1, the largest of the three C/EBPβ isoforms, led to a significant increase in colony formation when cells were grown in soft agar compared to empty vector transduced cells. In addition, depletion of C/EBPβ decreased colony formation, and re-expression of either C/EBPβ-1 or C/EBPβ-2 rescued the phenotype. We identified the cancer stem cell marker *ALDH1A1* as a target of C/EBPβ in Ewing sarcoma. Furthermore, increased expression of C/EBPβ led to resistance to chemotherapeutic agents. In summary, we have identified *CEBPB* as an oncogene in Ewing sarcoma. Overexpression of C/EBPβ-1 increases transformation, upregulates expression of the cancer stem cell marker ALDH1A1, and leads to chemoresistance.

## INTRODUCTION

Ewing sarcoma is the second most common bone cancer in children and young adults. While the cell of origin is not known, histologically it is comprised of characteristic small round blue cells. This disease has a poor outcome, with a 60% survival rate for localized disease and less than 20% survival for metastatic disease [1, 2]. Ewing sarcoma is defined by a translocation of the *EWSR1* gene to one of the ETS transcription factor family members, most commonly *FLI1*. This t(11;22) translocation encodes a fusion protein that acts as an oncogene by aberrantly transcribing genes involved in cellular proliferation and transformation [3, 4]. EWS-FLI1 binds with high affinity to GGAA microsatellite repeats within the promoter regions of its target genes [5, 6]. Investigation into the function of EWS-FLI1 target genes may help to identify disease risk factors and to develop novel treatments for Ewing sarcoma [7, 8].

We previously performed high resolution SNP microarray analysis with Molecular Inversion Probe (MIP) technology on 40 FFPE primary tumors from patients diagnosed with Ewing sarcoma in Utah [9]. We identified both known and novel regions of recurring copy number alterations (CNAs) and correlated our findings with clinical features including outcome. In our Utah cohort, chromosome 20q showed trisomy in 15% (*N* = 6/40) of tumors. This included 3 samples that had whole chromosome 20 gain, while 3 had chromosome 20q gain only. Of these three with chromosome 20q gain, one sample contained a very high gain (11 copies) within the 20q trisomy. This focal region in 20q13.13 was 575 kilobase pairs in length and centered on CCAAT/enhancer binding protein beta (*CEBPB*). Immunohistochemical staining showed increased C/EBPβ nuclear staining in this and other Ewing sarcoma samples with *CEBPB* gain compared to other Ewing sarcoma tumors and non-tumor controls with normal *CEBPB* copy number. The Ewing sarcoma sample with the high-gain of 11 copies showed the most intense IHC nuclear staining indicating increased C/EBPβ protein expression. Additionally, *CEBPB* gains correlated with worse outcome (EFS *P* = 0.012, OS *P* = 0.00013) [9]. Recent genomic landscape of Ewing sarcoma publications support our observation of trisomy in chromosome 20q in approximately 15% of Ewing sarcoma tumors [10, 11], suggesting that a copy number gain in this region may confer a survival disadvantage for these patients.

*CEBPB* encodes C/EBPβ, a leucine-zipper transcription factor involved in cellular metabolism, development, and differentiation [12–14]. Three protein isoforms of C/EBPβ (C/EBPβ-1, C/EBPβ-2, and C/EBPβ-3) are expressed through the use of alternate translational start sites [15]. These isoforms have distinct biological functions depending on the cellular context [16–19]. C/EBPβ is important for mesenchymal cell differentiation (a possible cell of origin for Ewing sarcoma) and promotes osteoblast differentiation [20, 21]. C/EBPβ also plays a role in promoting cellular proliferation and transformation in other cancer types, including skin cancer, breast cancer, and anaplastic lymphoma [12, 22, 23]. In addition, C/EBPβ expression levels are increased in a number of different tumor types [24]. While the literature supports a role for C/EBPβ in cancer and bone development, a C/EBPβ-driven mechanism in Ewing sarcoma has not yet been described. Our data indicate that C/EBPβ plays an oncogenic role in Ewing sarcoma and is regulated by the Ewing sarcoma causative translocation, EWS-FLI1.

C/EBPβ is a transcriptional regulator of aldehyde dehydrogenase 1A1 (ALDH1A1), a member of a family of detoxifying enzymes responsible for oxidizing aldehydes, in breast cancer cells [25]. ALDH is a proposed marker of cancer stem cells and ALDH activity has been used to identify cancer stem cells in breast, lung, and prostate cancer, among others [26–28]. Ewing sarcoma cells contain an ALDH-high population that are resistant to chemotherapy and have enriched sphere forming activity [29]. To our knowledge, our study is the first to explore the relationship between C/EBPβ and ALDH in Ewing sarcoma. Our data suggest that high levels of C/EBPβ lead to increased transformation, increased ALDH1A1 expression and activity, and chemotherapy resistance. Targeting ALDH-high cells in Ewing sarcoma may improve treatments in the future.

## RESULTS

### C/EBPβ is highly expressed in Ewing sarcoma

**Figure 1:**
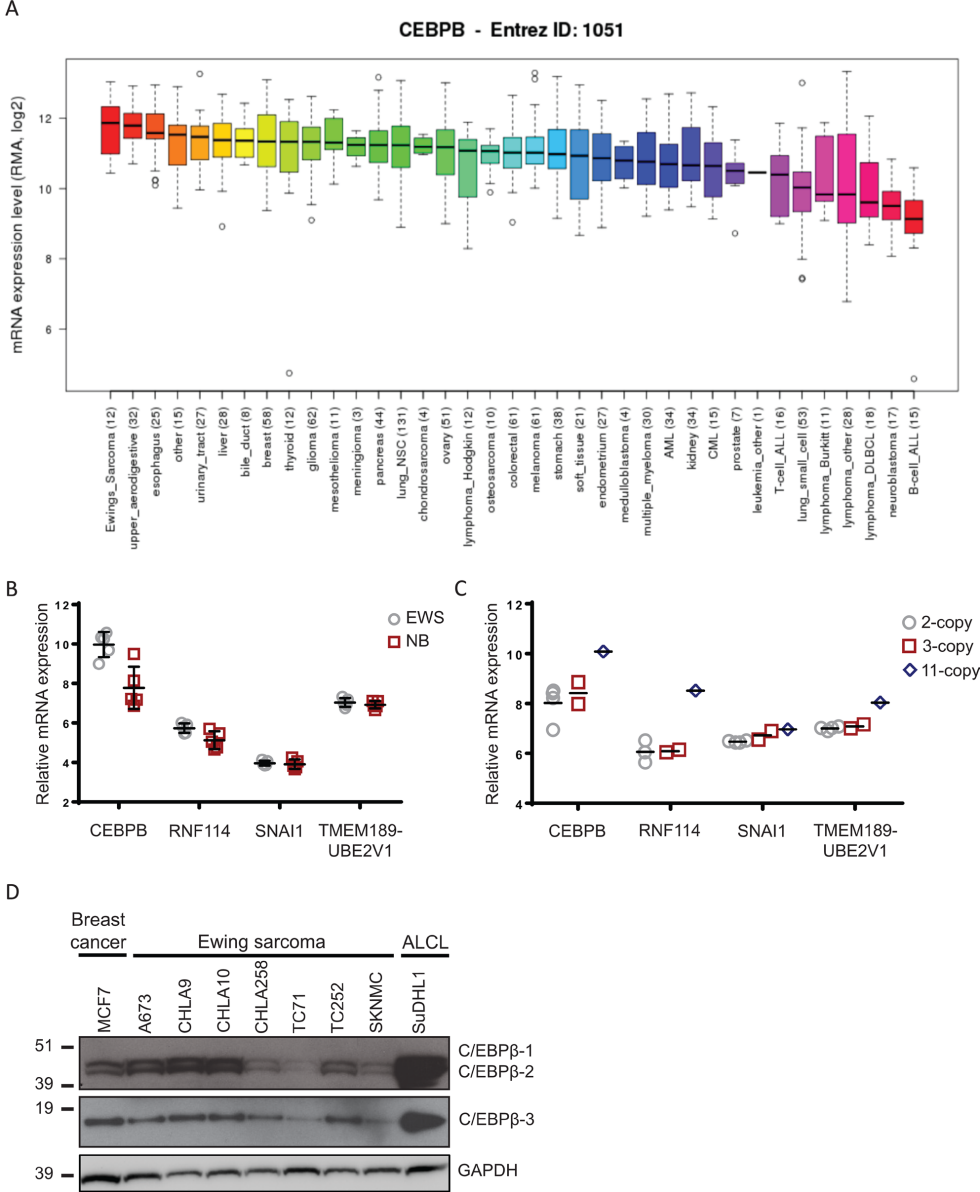
*CEBPB* expression in Ewing sarcoma (**A**) *CEBPB* expression was interrogated in cancer cell lines from the Cancer Cell Line Encyclopedia (Broad Institute). Ewing sarcoma cell lines had the highest *CEBPB* mRNA expression on average compared to any other cancer type. (**B**) Data from the GEO dataset GSE1825 was analyzed to determine relative mRNA expression of the genes within the region of high copy number gain on chromosome 20q in Ewing sarcoma patient samples compared to neuroblastoma patient samples (*CEBPB*: *P* = 0.016). (**C**) Relative mRNA expression of 4 genes within the region of gain on chromosome 20q with normal or increased copy number in Ewing sarcoma patient samples. (**D**) Expression of the C/EBPβ protein isoforms in Ewing sarcoma cell lines by Western blot. MCF7 (breast cancer) and SuDHL1 (anaplastic large cell lymphoma) were included as positive controls, and GAPDH serves as a loading control.

To determine if C/EBPβ is expressed in Ewing sarcoma cells, we interrogated the Broad Institute's Cancer Cell Line Encyclopedia (CCLE) [30] for expression of *CEBPB*. Out of the 37 different cancer types evaluated, Ewing sarcoma had the highest *CEBPB* expression on average (Figure 1A). Additionally, we evaluated the microarray dataset GSE1825 [31] from the GEO database for *CEBPB* expression in Ewing sarcoma patient samples compared to neuroblastoma patient samples and found significantly higher *CEBPB* expression in the Ewing sarcoma samples (*P* = 0.016) (Figure 1B).

Our initial study of CNAs in Ewing sarcoma identified a focal region of amplification in a subset of Ewing sarcoma tumors that contained five genes: *RNF114*, *SNAI1*, *UBE2V1*, *TMEM189*, and *CEBPB*. We used the same dataset (GSE1825) to evaluate expression of all five genes within the focal region of high copy gain in the Ewing sarcoma samples compared to neuroblastoma samples. Although *SNAI1* is a well-known gene involved in epithelial to mesenchymal transition in many cancers, it does not appear to be highly expressed in Ewing sarcoma tumors (Figure 1B). We further evaluated expression of these five genes in Ewing sarcoma tumor samples by microarray. Of the five genes, *CEBPB* mRNA expression levels in patient tumors corresponds most consistently with the number of copies of this region (Figure 1C), supporting the previously reported levels of protein expression by IHC [9].

Finally, a panel of Ewing sarcoma cell lines was screened for expression of each of the three protein C/EBPβ isoforms by Western blot. A breast cancer cell line (MCF7) and an anaplastic large cell lymphoma cell line (SuDHL1), previously reported to express C/EBPβ [23, 32], were included as positive controls (Figure 1D). Each of the 7 Ewing sarcoma cell lines (A673, CHLA9, CHLA10, CHLA258, TC71, TC252, and SKNMC) tested expressed all three protein isoforms of C/EBPβ in varying levels. Based on these results combined with the documented role of C/EBPβ in cancer and bone development, we decided to focus our efforts on understanding the functional role of C/EBPβ in Ewing sarcoma.

### Expression of C/EBPβ in Ewing sarcoma cells is controlled by EWS-FLI1

**Figure 2:**
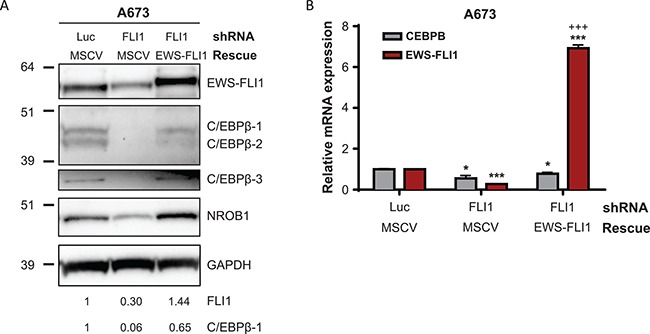
C/EBPβ expression in Ewing sarcoma cells is regulated by EWS-FLI1 (**A**) *EWS-FLI1* was knocked down with FLI1 shRNA and rescued in A673 cells. Luc is a non-targeting shRNA control and MSCV is an empty vector control. Expression of EWS-FLI1 and C/EBPβ protein isoforms was analyzed by Western blot, with GAPDH as a loading control. FLI1 and C/EBPβ bands were quantified by densitometry. (**B**) *EWS-FLI1* was knocked down and rescued in A673 cells. Relative *EWS-FLI1* and *CEBPB* transcript levels were measured by qRT-PCR. Multiple t tests were applied to determine statistical significance. (*P*-value for experimental vs control: *P < 0.05, **P < 0.005, ***P < 0.0001; *P*-value for experimental vs knockdown: ^+^*P* < 0.05, ^++^*P* < 0.005, ^+++^*P* < 0.0001.).

To determine whether the causative Ewing sarcoma translocation, EWS-FLI1, influences expression of C/EBPβ in Ewing sarcoma, we transduced a Ewing sarcoma cell line (A673) with a lentivirus expressing a shRNA targeting the 3’ untranslated region (3**′** UTR) of *FLI1*. This shRNA was previously validated and shown to specifically target *EWS-FLI1* in Ewing sarcoma cells [8]. Treatment with this shRNA led to a significant decrease in EWS-FLI1 protein and mRNA expression, as well as knockdown of protein and mRNA expression of all three C/EBPβ isoforms and NR0B1, a known target of EWS-FLI1 [7] (Figure 2A, 2B). To verify that this decrease in C/EBPβ expression was not due to an off-target effect by the shRNA, we transduced cells expressing *FLI* shRNA with a shRNA-resistant EWS-FLI1 expression construct to rescue expression of the fusion gene. Cells successfully transduced with both viruses were selected for 72 hours in puromycin (2 μg/ml) and G418 (500 μg/ml). Upon re-expression of EWS-FLI1, protein and mRNA expression of the C/EBPβ isoforms increased, indicating that C/EBPβ, like NR0B1, is regulated by EWS-FLI1 (Figure 2A, 2B). This is supported by publicly available EWS-FLI1 knockdown data in additional Ewing sarcoma cell lines [33, 34] (Supplementary Figure 1A, 1B). However, due to the lack of GGAA microsatellites in the *CEBPB* promoter region and EWS-FLI1 chIP-seq data generated by others [5, 6], we conclude that regulation of *CEBPB* by EWS-FLI1 is indirect and not mediated by direct binding of the fusion to the gene promoter.

### C/EBPβ does not affect cell proliferation and viability in Ewing sarcoma in 2D culture

**Figure 3:**
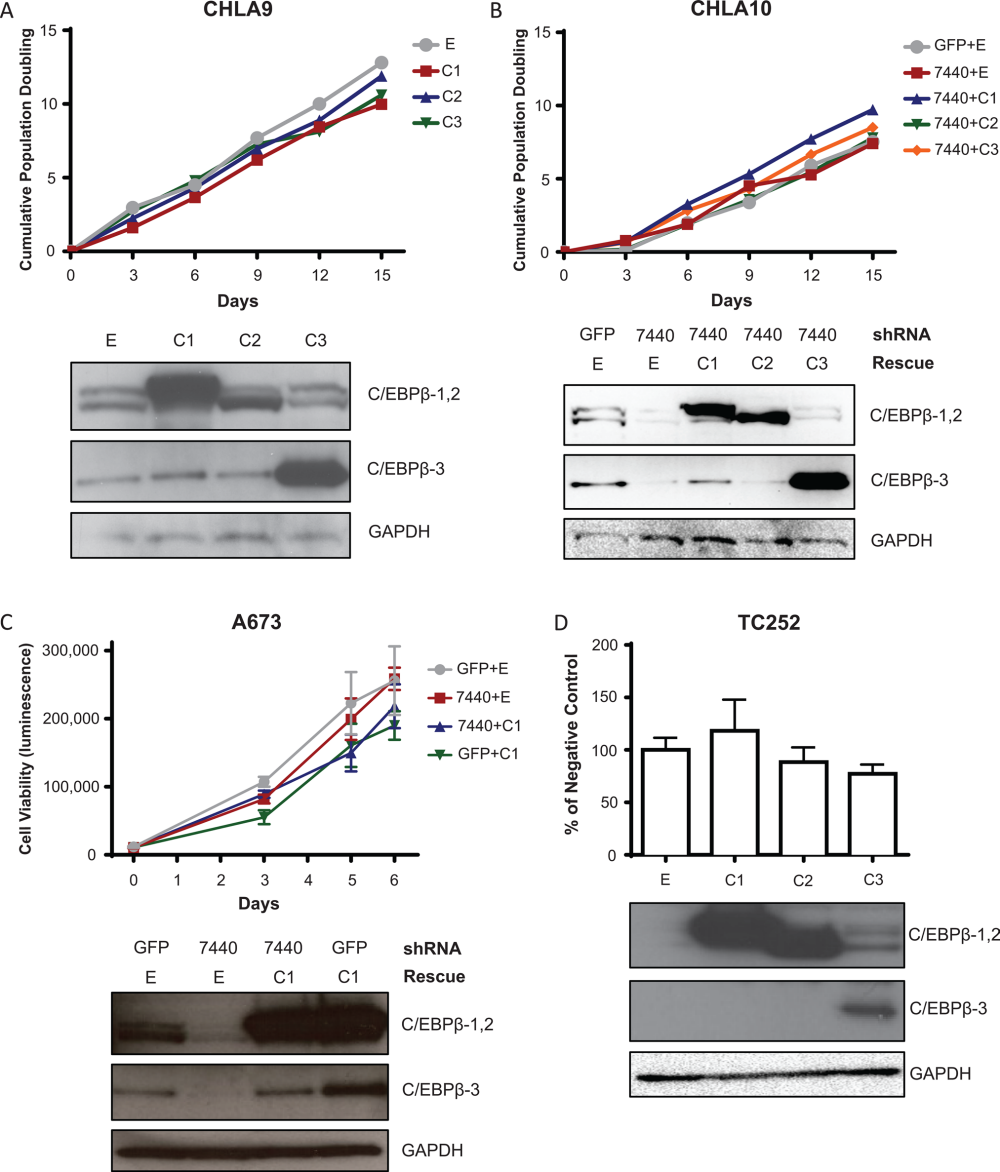
C/EBPβ isoform expression does not affect cell proliferation or viability in 2D culture (**A**) C/EBPβ isoforms were overexpressed in CHLA9 cells. Population doubling time was calculated by the 3T5 assay. (**B**) *CEBPB* was knocked down, then each isoform was rescued in CHLA10 cells. Population doubling time was calculated by the 3T5 assay. (**C**) *CEBPB* was knocked down then C/EBPβ-1 was rescued in A673 cells. Cell viability was measured over time with Cell Titer Glo (Promega) with raw luminescence values representing cell viability. (**D**) *CEBPB* isoforms were overexpressed in TC252 cells. Cell viability was measured 72 hours after cells were plated. Multiple *t* tests demonstrate no statistical difference between treatments and control. Western blots in all panels show C/EBPβ isoform expression, with GAPDH included as a protein loading control. E = empty vector (control); C1 = C/EBPβ-1; C2 = C/EBPβ-2; C3 = C/EBPβ-3.

To evaluate the functional consequences of C/EBPβ isoform expression in Ewing sarcoma, each of the three C/EBPβ isoforms were expressed individually in Ewing sarcoma cell lines by retroviral transduction. To determine which isoforms, if any, affect the rate of cell proliferation in standard culture conditions (2D culture), we overexpressed each of the C/EBPβ isoforms in Ewing sarcoma cell lines and calculated cell doubling time by the 3T5 assay [35]. There was no difference in cell doubling time with overexpression of any of the three isoforms compared to the empty vector control (Figure 3A). Similarly, when *CEBPB* was knocked down with shRNA that targets the 3′UTR of *CEBPB* (7440; Supplementary Figure 2A) there was no significant decrease in cell proliferation, and there was no significant increase in cell proliferation with rescue of any of the C/EBPβ isoforms (Figure 3B, 3C). Additionally, we measured no difference in cell viability over time with *CEBPB* knockdown and rescue, or after 72 hours following overexpression of the different C/EBPβ isoforms (Figure 3C, 3D). These results suggest that C/EBPβ expression does not affect cell proliferation and viability in Ewing sarcoma cells in 2D culture.

### C/EBPβ overexpression promotes transformation in Ewing sarcoma cells

**Figure 4:**
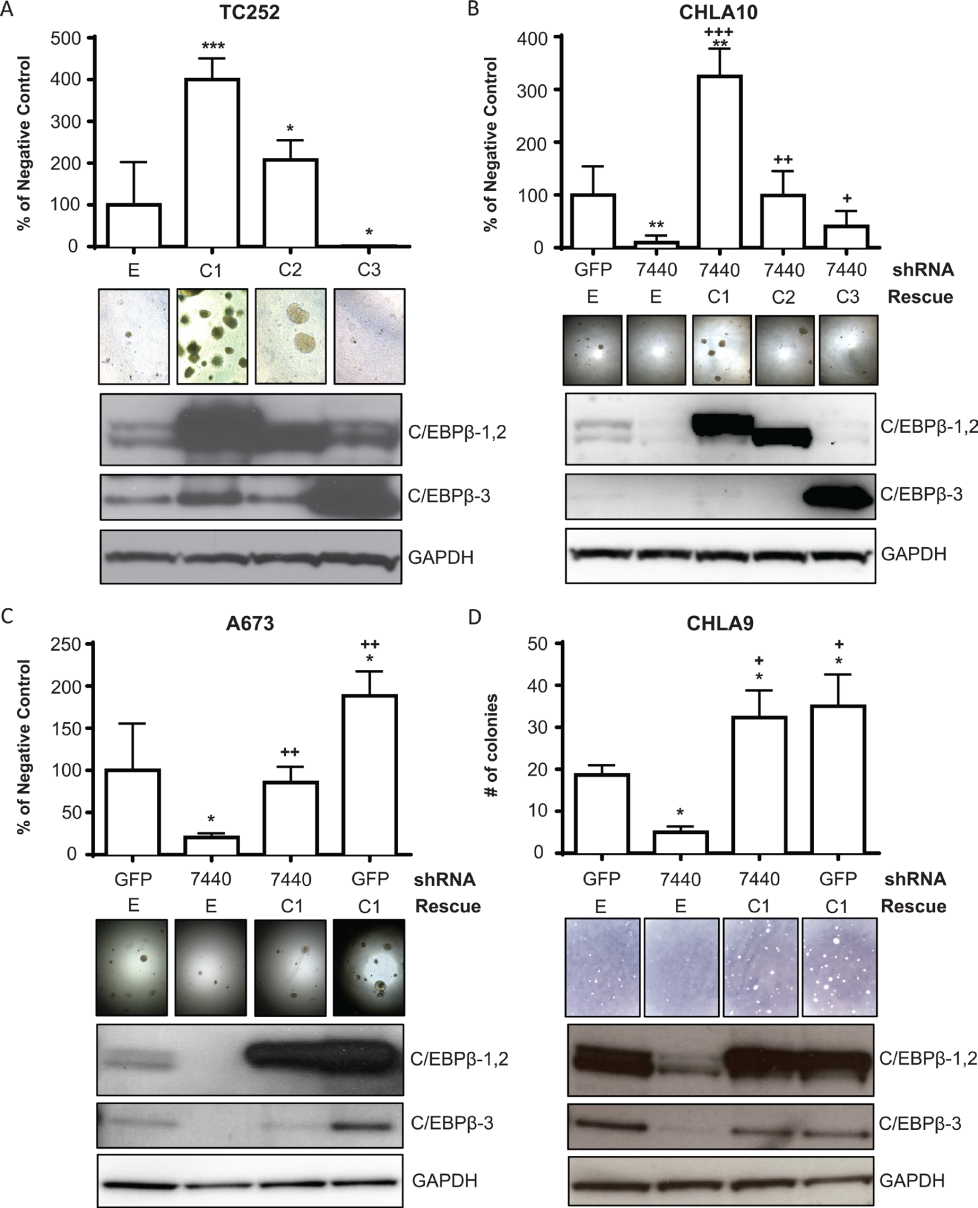
C/EBPβ-1 promotes attachment-independent cellular transformation (**A**) C/EBPβ isoforms were overexpressed in TC252 cells and grown in soft agar. Colonies were imaged and cell viability was measured after 14 days. (**B**) C/EBPβ was depleted, then each isoform was rescued in CHLA10 cells. Cells were grown in soft agar and colonies were imaged and cell viability measured after 16 days. (**C**) C/EBPβ was depleted and C/EBPβ-1 was rescued or overexpressed in A673 cells. Cells were grown in soft agar and colonies were imaged and cell viability was measured after 14 days. (**D**) C/EBPβ was depleted and C/EBPβ-1 was rescued or overexpressed in CHLA9 cells. Cells were grown in soft agar and colonies were imaged and counted after 27 days. Protein expression of each of the three overexpressed, knocked down, or rescued isoforms in each panel is shown. GAPDH is included as a loading control. Multiple t tests were performed to determine statistical difference between treatments and control in each experiment. (*P*-value for experimental vs control: *P < 0.05, **P < 0.005, ***P < 0.0001; *P*-value for experimental vs knockdown: ^+^*P* < 0.05, ^++^*P* < 0.005, ^+++^*P* < 0.0001). E = empty vector (control); C1 = C/EBPβ-1; C2 = C/EBPβ-2; C3 = C/EBPβ-3.

To determine the effect of C/EBPβ expression on cellular transformation, Ewing sarcoma cell lines were transduced to express each of the three C/EBPβ isoforms individually. Following antibiotic selection, cells were grown in attachment-independent conditions (soft agar) for 2–4 weeks, and cell viability was measured and colonies were counted. We observed an increase in cell viability, colony number, and colony size with overexpression of C/EBPβ-1 and C/EBPβ-2 (Figure 4). Conversely, there was a decrease in colony formation with knockdown of *CEBPB* expression compared to treatment with a non-targeting shRNA control (GFP) (Figure 4B–4D; Supplementary Figure 2A). To rule out the possibility that the decrease in colony formation is due to shRNA off-target effects, C/EBPβ expression was rescued by transducing C/EBPβ knockdown or control (GFP) knockdown cells with retroviruses encoding each C/EBPβ isoform. The colony-formation phenotype was rescued with overexpression of C/EBPβ-1 and C/EBPβ-2 following knockdown (Figure 4B–4D), suggesting that C/EBPβ-1 and C/EBPβ-2 expression increases transformation of Ewing sarcoma cells.

### *ALDH1A1* is a target of C/EBPβ

**Figure 5:**
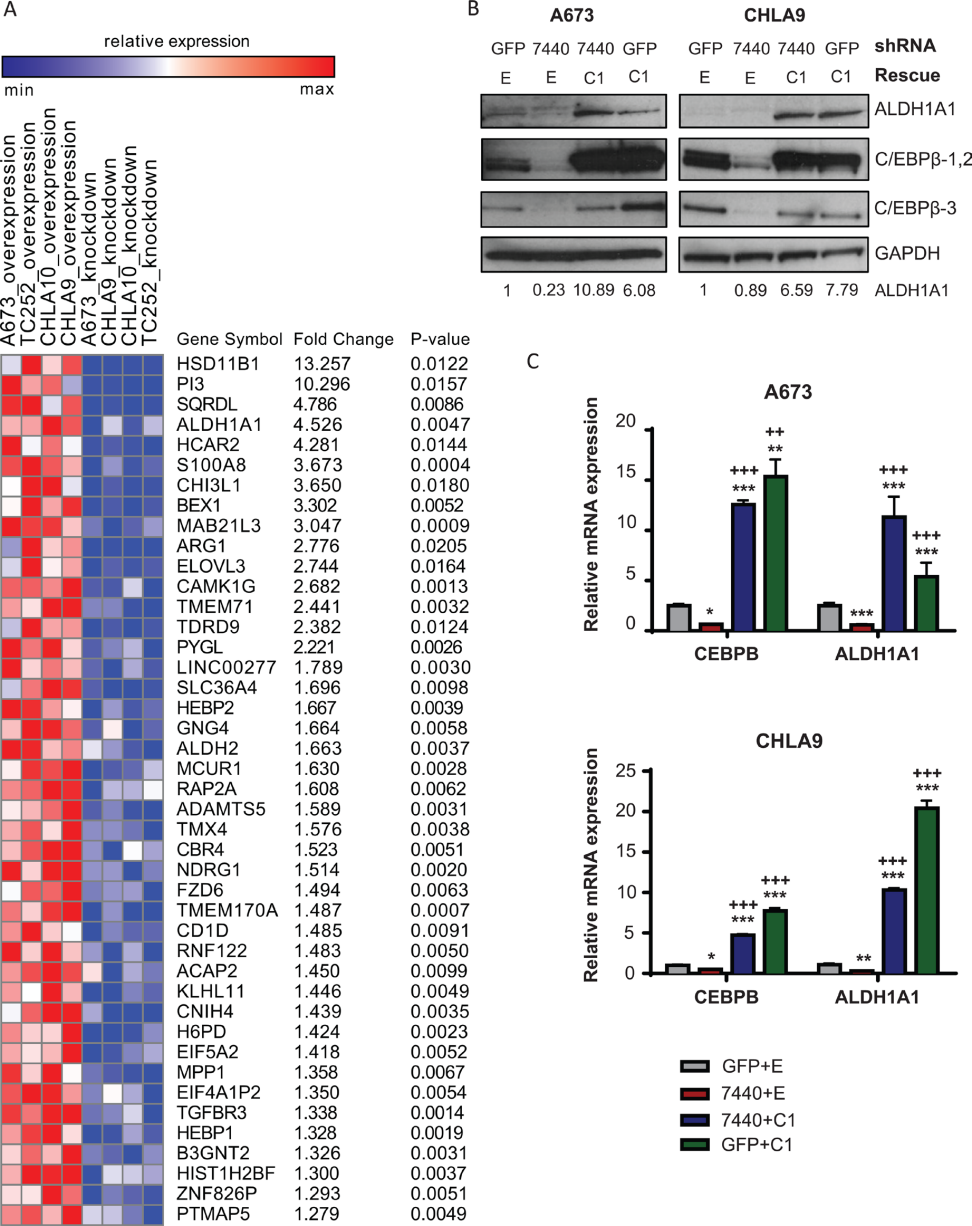
*ALDH1A1* is a target of C/EBPβ-1 (**A**) Heat map of differentially expressed genes between C/EBPβ-1 knockdown and C/EBPβ-1 overexpression normalized to control in four Ewing sarcoma cell lines. Gene list was ranked by average fold-change between C/EBPβ-1 knockdown and overexpressed samples normalized to control sample in each cell line. Top 43 genes (fold change > 1.25; permutation test *P* < 0.05) that positively correlated to C/EBPβ-1 expression are shown on the list. *P*-values by t-test for all genes included on the heat map are listed. (**B**) ALDH1A1 protein expression was analyzed by Western blot in A673 and CHLA9 cells with C/EBPβ knockdown, rescue, and overexpression. ALDH1A1 bands were quantified by densitometry. (**C**) Relative expression of *CEBPB* and *ALDH1A1* mRNA in the same cells as in B. Expression was normalized to endogenous *GAPDH*. Multiple t tests were performed to determine statistical significance among treatments. (*P*-value for experimental vs control: *P < 0.05, **P < 0.005, ***P < 0.0001; *P*-value for experimental vs knockdown: ^+^*P* < 0.05, ^++^*P* < 0.005, ^+++^*P* < 0.0001). E = empty vector (control); C1 = C/EBPβ-1; C2= C/EBPβ-2; C3= C/EBPβ-3.

We explored potential downstream targets of C/EBPβ by microarray in four Ewing sarcoma cell lines (A673, CHLA9, CHLA10, TC252) harboring altered C/EBPβ-1 expression: C/EBPβ lentiviral knockdown; C/EBPβ-1 retroviral overexpression; C/EBPβ-1 rescue by lentiviral knockdown followed by retroviral overexpression; empty vector and non-targeting shRNA control treatment; and untreated cells (Supplementary Figure 3A, 3C). RNA from these cells was run on the Human Transcriptome Array 2.0 (Affymetrix). There were 43 genes that had greater than 1.25 fold change and *P*-value < 0.05 (permutation test) between C/EBPβ-1 knockdown and C/EBPβ-1 overexpression. *HSD11B1*, *PI3*, *SQRDL*, *ALDH1A1*, and *HCAR2* were the top 5 most differentially expressed genes when comparing normalized C/EBPβ-1 depleted cells with normalized C/EBPβ-1 overexpressing cells in all four cell lines (Figure 5A, Supplementary Table 1).

Because of its role as a cancer stem cell marker and its involvement in transformation and chemoresistance [26–29], we directed our focus to understanding ALDH1A1 in Ewing sarcoma and its relationship to C/EBPβ. Additionally, previous reports have demonstrated the presence of a CCAAT box in the promoter region of ALDH1A1 where C/EBPβ interacts to promote transcription [25, 36]. To validate the array data, ALDH1A1 protein expression was measured by Western blot in Ewing sarcoma cell lines with C/EBPβ-1 depletion, rescue, and overexpression. We observed an increase in ALDH1A1 protein expression with C/EBPβ-1 overexpression and a decrease in ALDH1A1 protein expression with C/EBPβ-1 knockdown (Figure 5B). We further measured *CEBPB* and *ALDH1A1* transcript levels in these cells by qRT-PCR and observed a similar trend: *ALDH1A1* transcript levels positively correspond to *CEBPB* levels (Figure 5C; Supplementary Figure 3B, 3C). This suggests that C/EBPβ functions as a transcriptional regulator of ALDH1A1 in Ewing sarcoma.

### C/EBPβ overexpressing cells have high ALDH activity

**Figure 6:**
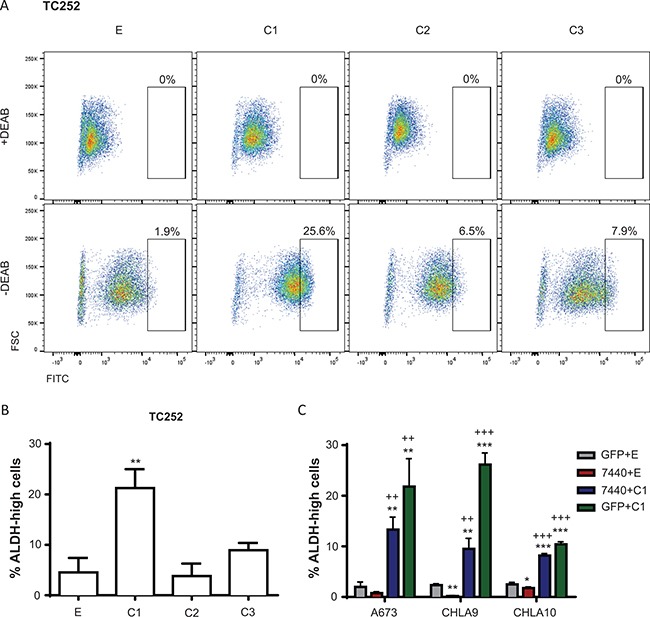
ALDH activity is influenced by C/EBPβ expression (**A**) Representative FACS analysis of TC252 cells subject to the Aldefluor Assay. Percentage of viable cells that are gated as ALDH-high (FITC positivity) is shown. (**B**) Percentage of live TC252 cells from A that are gated as ALDH-high with overexpression of each C/EBPβ isoform. (**C**) C/EBPβ was depleted, rescued, and overexpressed in A673, CHLA9, and CHLA10 cells, and cells were subject to the Aldefluor Assay. Percentage of live cells that are gated as ALDH-high in each condition is shown. Multiple *t* tests were used to determine statistical significance. (*P*-value for experimental vs control: *P < 0.05, **P < 0.005, ***P < 0.0001; *P*-value for experimental vs knockdown: ^+^*P* < 0.05, ^++^*P* < 0.005, ^+++^*P* < 0.0001). E = empty vector (control); C1 = C/EBPβ-1; C2 = C/EBPβ-2; C3 = C/EBPβ-3.

A subpopulation of Ewing sarcoma cells express high levels of ALDH, and this ALDH-high population has been shown to have stem cell like properties, increased colony formation, and increased chemoresistance compared to ALDH-low Ewing sarcoma cells [29]. Based on this previous report, we explored the possibility that increased expression of C/EBPβ leads to increased activity of ALDH. Ewing sarcoma cells overexpressing the C/EBPβ isoforms were subject to the Aldefluor assay to measure ALDH activity. Cells were treated with diethylaminobenzaldehyde (DEAB), an ALDH inhibitor, as a negative control. ALDH activity was measured through the Aldefluor assay by flow cytometry, where FITC-positive cells represent high ALDH activity. Cells overexpressing C/EBPβ-1 had a greater population of ALDH-high cells, suggesting these cells have greater ALDH activity compared to controls and cells overexpressing C/EBPβ-2 or -3 (*P* < 0.005) (Figure 6A, 6B). When C/EBPβ-1 was knocked down in Ewing sarcoma cells, there was a decrease in ALDH activity; and ALDH activity increased with rescued expression of C/EBPβ-1 (Figure 6C; Supplementary Figure 4). This indicates that C/EBPβ-1 overexpressing cells not only have high ALDH1A1 levels, but high ALDH activity.

### C/EBPβ-1 overexpressing cells are resistant to chemotherapies

**Figure 7:**
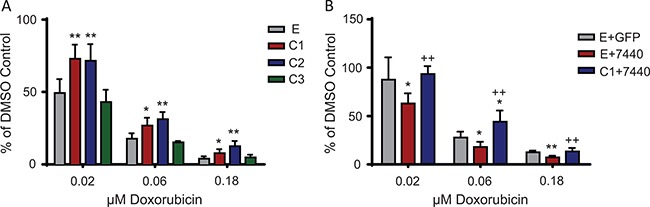
C/EBPβ overexpression leads to chemoresistance in Ewing sarcoma cells (**A**) Each of the C/EBPβ isoforms were overexpressed in TC252 cells. Cells were treated with the indicated doses of doxorubicin for 48 hours, then viability was measured. (**B**) C/EBPβ was depleted, then C/EBPβ-1 was rescued in TC252 cells. Cells were treated with doxorubicin for 48 hours, then cell viability was measured. Multiple *t* tests were performed to determine statistical difference between treatments and controls. (*P*-value for experimental vs control: *P < 0.05, **P < 0.005, ***P < 0.0001; *P*-value for experimental vs knockdown: ^+^*P* < 0.05, ^++^*P* < 0.005, ^+++^*P* < 0.0001). E = empty vector (control); C1 = C/EBPβ-1; C2 = C/EBPβ-2; C3 = C/EBPβ-3.

To evaluate whether C/EBPβ expression affects the cellular response to chemotherapies, Ewing sarcoma cells transduced to express each isoform of C/EBPβ were treated with doxorubicin, part of the regular chemotherapeutic regimen for Ewing sarcoma patients. Cells grown in normal, attachment-dependent conditions, overexpressing either C/EBPβ-1 or C/EBPβ-2, had significantly increased viability after 48 hours of doxorubicin treatment compared to control cells (empty vector) and C/EBPβ-3 overexpressing cells (*P* < 0.05) (Figure 7A; Supplementary Figure 5A). Additionally, there was a decrease in cell viability after 48 hours of doxorubicin treatment when C/EBPβ was depleted by shRNA; and viability returned to control levels with rescued expression of C/EBPβ-1 (Figure 7B). These results suggest that Ewing sarcoma cells with increased expression of C/EBPβ-1 or C/EBPβ-2 are more chemoresistant than cells without.

## DISCUSSION

To explore the function of C/EBPβ isoforms in Ewing sarcoma, we overexpressed each of the three C/EBPβ isoforms in Ewing sarcoma cell lines. Overexpression of C/EBPβ-1 and C/EBPβ-2 resulted in significant increases in cellular transformation as measured by colony formation in soft agar. Interestingly, in breast cancer, C/EBPβ-2 is the isoform capable of transforming normal mammary epithelial cells [22]. In Ewing sarcoma cells, both C/EBPβ-1 and C/EBPβ-2 increase transformation, yet the increase in colony formation is consistently greater with overexpression of C/EBPβ-1 compared to C/EBPβ-2. C/EBPβ-1 protein was more highly expressed compared to C/EBPβ-2 in each cell line tested, which may account for the difference in colony formation observed between the two isoforms.

Cellular context appears to play an important role in the Ewing sarcoma cell response to increased expression of the C/EBPβ isoforms, particularly C/EBPβ-3. In some cell lines, C/EBPβ-3 inhibits colony formation (Figure 4A, 4B), while C/EBPβ-3 overexpression in other cell lines increases colony formation (data not shown). Likewise, C/EBPβ-3 overexpression in TC252 did not confer resistance to doxorubicin, while C/EBPβ-3 overexpression in CHLA10 did (Figure 7A, Supplementary Figure 5A). This difference may result from variation in the level of C/EBPβ-3 expression in each cell line. Additionally, these differences may be due to the ratio of expression of each isoform, as C/EBPβ-3 is capable of antagonizing the larger isoforms in certain contexts [15]. Previous studies have shown the importance of C/EBPβ isoform ratios in bone development and cell cycle progression in the liver [16, 37]. However, since both cell lines express a high level of this isoform, we favor the alternative possibility that differences in cellular context lead to the observed variation in colony formation and drug resistance. These two cell lines may also express varying levels of downstream targets of C/EBPβ, which would affect their response to C/EBPβ expression in a cell line dependent manner.

The retroviral expression system used in this study did not allow us to control the level of C/EBPβ expression. The level of expression in these *in vitro* experiments may not accurately reflect the amount of increased protein expression in patient cells. However, even if the amount of C/EBPβ expressed in our transduced cell lines was greater than that of patient tumors, knockdown of C/EBPβ expression significantly decreased colony formation and increased chemosensitivity, further supporting a critical role for this gene in Ewing sarcoma transformation and growth.

In an effort to better understand how C/EBPβ upregulation increases transformation potential in Ewing sarcoma, we sought to identify downstream targets of C/EBPβ. By microarray analysis, we identified aldehyde dehydrogenase 1A1 (*ALDH1A1*) as a target of C/EBPβ. In breast cancer cell lines, C/EBPβ-2 has been shown to regulate *ALDH1A1* expression [25]. ALDH1A1 is an aldehyde dehydrogenase involved in alcohol metabolism and oxidation of cellular aldehydes. High expression of ALDH1A1 is found in many cancers and is frequently considered a marker of cancer stem cells [28, 29, 38]. C/EBPβ-1 overexpressing cells had high transcript and protein levels of ALDH1A1, as well as high ALDH activity. Furthermore, overexpression of C/EBPβ-1 and C/EBPβ-2 render Ewing sarcoma cells more resistant to doxorubicin. This resistance may be mediated in part by upregulation of ALDH, as previous studies have shown ALDH-high cells to be more resistant to chemotherapies compared to ALDH-low cells, though the mechanism is not well-understood and warrants further investigation [29]. ALDH1A1 may serve as a biomarker for treatment resistance in Ewing sarcoma patients.

Taken together, these results support the oncogenic role of C/EBPβ-1 in Ewing sarcoma. The increase in cellular transformation and growth with C/EBPβ-1 overexpression in cell lines may explain the poor outcome of patients previously reported to have *CEBPB* copy number gain. As a transcriptional target of EWS-FLI1, the causative translocation of the majority of Ewing tumors, *CEBPB* is likely up-regulated in many cases of Ewing sarcoma. Further, as a transcription factor itself, C/EBPβ regulates the expression of downstream targets, such as *ALDH1A1*, a potential cancer stem cell marker in Ewing sarcoma and a superior therapeutic target. Understanding the molecular contributions of C/EBPβ to ALDH1A1 and other downstream targets in Ewing sarcoma warrants further investigation.

## MATERIALS AND METHODS

### Cell culture

A673 (American Type Culture Collection), CHLA9 and CHLA10 (Children's Oncology Group Cell Culture and Xenograft Repository), and TC252 were used in this study. A673 cells were cultured in DMEM supplemented with 10% fetal bovine serum, 1% glutamax, and 1% sodium pyruvate. TC252 cells were cultured in RPMI supplemented with 10% fetal bovine serum and 1% glutamax. CHLA9 and CHLA10 cells were cultured in IMDM supplemented with 20% fetal bovine serum, 2% glutamax, and 1% insulin-transferrin-selenium. All experiments were performed within 6 months of cell line resuscitation, and cell line authentication was performed by providers by STR profiling.

### Cloning

*CEBPB* was cloned from an Origene Human cDNA clone (SC319561, Origene; accession number NM_005194.2). The following primers were used to amplify *CEBPB*: forward primer for *CEBPB-1* - 5′- GTC CGG AAT TCG CCG CCG CCATGC AAC GCCTGGTGG CCT G -3′, forward primer for *CEBPB-2* - 5′- GTC CGG AAT TCG CCG CCG CCATGG AAGTGG CCA ACT TCT ACT ACG AGG CG -3′, and forward primer for *CEBPB-3* - 5′- GTC CGG AAT TCG CCG CCG CCATGG CGG CGG GCT TCC CGT ACG -3′. The same reverse primer was used to clone all three isoforms: 5′- GAATTA AGATCT CTA GCA GTG GCC GGA GGA GGC GA -3′. These primers add restriction sites for cloning and a Kozak consensus sequence for translation. Platinum Pfx DNA Polymerase kit (Life Technologies) with enhancer was used to amplify each isoform. Product sizes were verified by running the samples on 1.5% agarose gels and visualizing bands with UV light. Bands of the appropriate size were cut from the gels and purified using the QIAquick Gel Extraction Kit (Qiagen). Next, the PCR products and empty vector MSCVneo (Clontech) were digested with EcoRI and BglII. Digested vector and inserts were gel purified as above, followed by ligation with T4 DNA Ligase (Life Technologies). Stbl3 (Life Technologies) chemically competent bacteria were transformed by heat shock. Four colonies from each transformation were screened after miniprep (Qiagen Miniprep Kit) by digesting with EcoRI and BglII. Plasmids with inserts of the appropriate size were confirmed by Sanger sequencing (University of Utah DNA Sequencing Core).

### Viral packaging

Five *CEBPB* MISSION shRNA Plasmid DNA (Sigma) clones were used in knockdown experiments (TRCN0000007440, TRCN0000007441, TRCN0000007442, TRCN0000007443, TRCN0000007444). shRNA lentivirus was packaged in HEK293 cells using Sigma Mission Lentiviral Packaging mix. For overexpression, MSCV constructs containing *CEBPB* were packaged into retroviruses by co-transfection with packaging plasmids in 293-EBNA (Life Technologies) cells.

### Viral transduction of cell lines

300,000-500,000 Ewing sarcoma cells were plated in 6-well culture plates. Following adherence of cells (6–24 hours), lentivirus or retrovirus was added along with 10 μg/ml Polybrene (Millipore). After 24 hours in culture, media containing virus was removed and media containing antibiotic was added. Cells were cultured for at least 72 hours in 2 μg/ml puromycin or 120 hours in 500 μg/ml G418 to select for successfully transduced cells. Experiments were carried out in the presence of antibiotic.

### Western Blot

Cells were lysed in Cell Lysis Buffer (Cell Signaling Technology) supplemented with Protease/Phosphatase Inhibitor Cocktail (Cell Signaling Technology). 30–50 μg of total protein lysate was run on Bolt 10% Bis-Tris Plus or NuPAGE 7% Tris-Acetate gels (Life Technologies) and transferred to PVDF membranes (Life Technologies). Membranes were blocked in StartingBlock T20 (TBS) blocking buffer (Thermo Scientific), and proteins were detected with the following antibodies: rabbit monoclonal Anti-C/EBP-β (Clone E299, Millipore), rabbit polyclonal Anti-FLI1 (ab15289, Abcam), rabbit polyclonal Anti-DAX1 (also known as NR0B1, sc-841, Santa Cruz Biotechnology), rabbit monoclonal Anti-ALDH1A1 (EP1933Y, Abcam), or mouse monoclonal Anti-GAPDH (Clone GAPDH-71.1, Sigma Aldrich) followed by HRP-anti-rabbit or HRP-anti-mouse secondary (GE Healthcare Life Sciences). Substrates used for detection were SuperSignal West Dura Chemiluminescent Substrate (Thermo Scientific) and Western Lightning Ultra (PerkinElmer). Signal was detected with a Bio-Rad ChemiDoc Gel Imager or developed on Amersham Hyperfilm ECL (GE Healthcare Life Sciences) or HyBlot CL Autoradiography Film (Denville Scientific). Bands were quantified using ImageJ.

### qRT-PCR

RNA was analyzed by qPCR following the EXPRESS qPCR SuperMix Universal protocol (Invitrogen) on a StepOne Plus qPCR machine (Applied Biosystems). The following primer/probes were used: EWS-FLI1 Hs03024807_ft, CEBPB Hs00270923_s1, GAPDH Hu GAPDH 4310884E-1003044, and ALDH1A1 Hs00946916_m1 (all from Applied Biosystems). Samples were run in triplicate and normalized to endogenous *GAPDH* expression.

### 3T5 assay

3T5 Assay was performed as previously reported [35]. Briefly, 500,000 cells were plated in 10-cm dishes and grown under normal conditions for 3 days. Cells were counted, and 500,000 cells plated in new 10-cm dish. Process repeated every 3 days for 15 days.

### Colony formation and cell viability assay

Following viral transduction and antibiotic selection, cells were seeded at 5000 cells/well in triplicate in black clear-bottom 96-well plates. Anchorage-dependent cell viability was measured with 1× Cell Titer Glo (Promega) prior to culture to confirm equal seeding density and after the indicated amount of time in culture. For the soft agar colony assay, 50 μl/well of 0.8% Lonza SeaPlaque GTG agarose in appropriate media for cell type plus selective antibiotic was plated in replicates of 6 in black clear-bottom 96-well plates. After the bottom layer solidified, cells were seeded at 500–1500 cells/well in 0.4% Lonza SeaPlaque GTG agarose in appropriate media (supplemented with antibiotic) on top of the bottom layer. 100 μl of media containing antibiotic was added on top of the cell layer following solidification. Colonies were imaged at 4×-10× magnification and cell viability was measured with Cell Titer Glo, 14–28 days after seeding. Soft agar assays were also conducted in 12-well plates. For these, 600 μl/well of 0.8% Lonza SeaPlaque GTG agarose in media appropriate for cell type was added to wells of 12-well plate and allowed to solidify at room temperature. Cells were then seeded at 250–1000 cells/well in 0.4% Lonza SeaPlaque GTG agarose in appropriate media on top of bottom layer and allowed to solidify. 500 μl of media containing selective antibiotic was added on top of the cell layer. Colonies were imaged and counted using ImageJ 2–4 weeks after seeding.

### RNA extraction

FFPE tumor ribbons from patients with Ewing sarcoma were deparaffinized with Hemo-De, tissue was washed twice in 100% ethanol, and allowed to air dry for 45 minutes. Tissue was digested and RNA was isolated following the Recoverall Total Nucleic Acid Isolation protocol (Ambion Life Technologies). RNA was isolated from cell lines using QIAGEN's RNeasy Mini protocol with QIAshredder (QIAGEN). RNA was treated with DNase using the TURBO DNA-*free* Kit (Ambion Life Technologies) and quantified using Quant-iT Ribogreen RNA Reagent and Kit (Invitrogen).

### Drug treatment

Ewing sarcoma cells transduced with *CEBPB* isoforms were seeded at 1000 cells/well in black clear-bottom 96-well tissue culture plates. 24 hours following seeding, doxorubicin was added at the indicated concentrations. DMSO was used as a no-treatment control (doxorubicin stock was dissolved in DMSO). Cell viability was measured with Cell Titer Glo (Promega) 48 hours following drug treatment.

### Gene expression analysis

RNA from each condition in each cell line (A673, CHLA9, CHLA10, TC252) was collected and processed using the SensationPlus™ FFPE Amplification and WT Labeling Kit, optimized for FFPE samples, according to the manufacturer's protocol (Affymetrix, Santa Clara, CA). Briefly, 50 ng of total RNA was used for first strand cDNA synthesis using a random/dT primer mix. Addition of a poly A tail and T7 promoter region allowed for *in vitro* transcription of senseRNA. The senseRNA was then reverse transcribed to produce double stranded cDNA, which was fragmented, labeled and hybridized to the human transcriptome array (HTA) 2.0 (Affymetrix, Santa Clara, CA). Raw Affymetrix CEL files were processed by Affymetrix Expression Console (1.4.1) at the gene level and exon level. Background correction and normalization were done by the RMA-Sketch algorithm. The processed data were exported in a matrix that contains log_2_ intensity values for each probe set. For each gene, the levels in C/EBPβ overexpression or knockdown treatments were normalized to the level of control in each cell line. Normalized data were used to generate a heat map of differentially expressed genes by GENE-E (Broad). Samples were grouped by the condition of C/EBPβ (normalized to control): overexpression or knockdown. The final gene list was ranked based on fold-changes and includes the genes with fold-change of overexpression to knockdown > 1.25 and *P*-value < 0.05 (permutation test). No multiple comparisons tests were performed due to our small sample size. Individual gene *P*-values of the most differentially expressed genes were calculated using the *t*-test.

### Aldefluor assay

The Aldefluor Assay (Stem Cell Technologies) was performed on Ewing sarcoma cells according to the manufacturer's protocol. Cells were analyzed by flow cytometry using a BD FACSCanto Analyzer and FlowJo software. Viable cells were gated based on propidium-iodide exclusion. ALDH-high cells were quantified by gating the top 2% of FITC+ empty vector control cells.

### Statistical analysis

Multiple *t* tests were applied to analyze statistical differences among treatments, unless otherwise indicated. Asterisks in the figures indicate significance to the empty vector (negative) control (*P < 0.05, **P < 0.005, ***P < 0.0001) and plus signs in the figures indicate significance to the knockdown (^+^*P* < 0.05, ^++^*P* < 0.005, ^+++^*P* < 0.0001).

## SUPPLEMENTARY MATERIALS FIGURES AND TABLE


