# Supplementary material for: C/EBPβ-1 promotes transformation and chemoresistance in Ewing sarcoma cells
Source: Oncotarget. 2017 Jan 27;8(16):26013–26. doi: 10.18632/oncotarget.14847 (PMC5432234; doi:10.18632/oncotarget.14847)
Supplement: Supplementary file 1 [file oncotarget-08-26013-s001.pdf]

C/EBPβ-1 promotes transformation and chemoresistance in Ewing sarcoma cells

Supplementary Materials

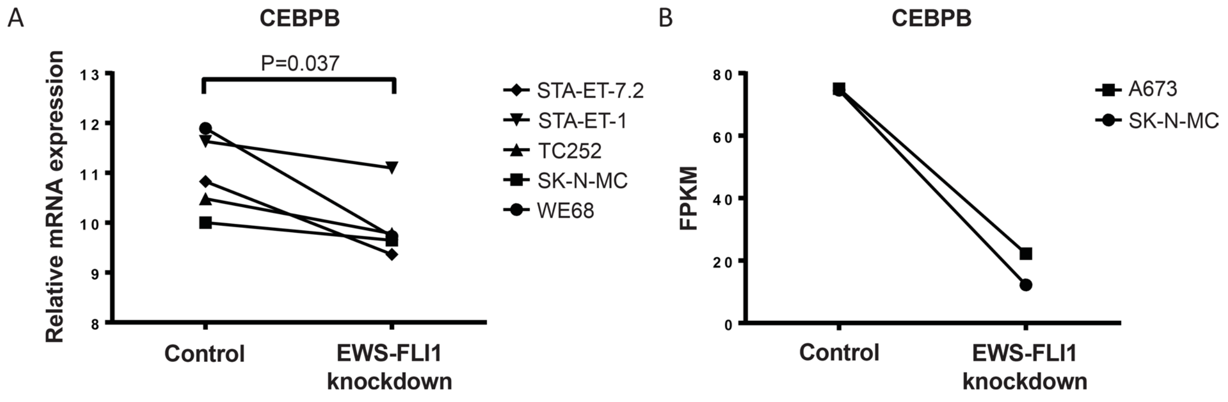

Supplementary Figure 1: *CEBPB* expression decreases with EWS-FLI1 knockdown. (**A**) *CEBPB* expression in Ewing sarcoma cell lines with or without EWS-FLI1 knockdown. Data was analyzed from Kauer et al. 2009 [33]. (**B**) *CEBPB* expression in Ewing sarcoma cell lines with or without EWS-FLI1 knockdown. Data was analyzed from Riggi et al. 2014 [34].

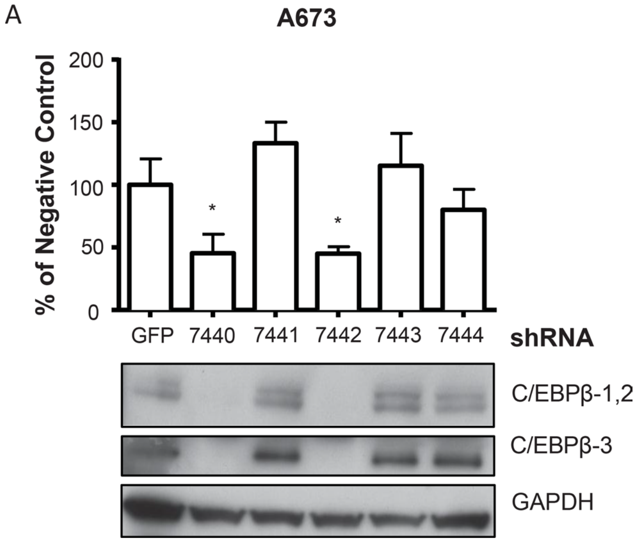

Supplementary Figure 2: Depletion of C/EBPβ by shRNA. (**A**) A panel of 5 shRNAs were screened for successful C/EBPβ protein knockdown. shRNAs 7440 and 7442 sufficiently knocked down all C/EBP β isoforms. GFP was included as a non-targeting shRNA control. Cells were grown in soft agar for 12 days, then viability was measured and is shown in the graph. Multiple t tests were performed to determine statistical significance between knockdown and control. (P-value for experimental vs control: $*P < 0.05$, $**P < 0.005$, $***P < 0.0001$).

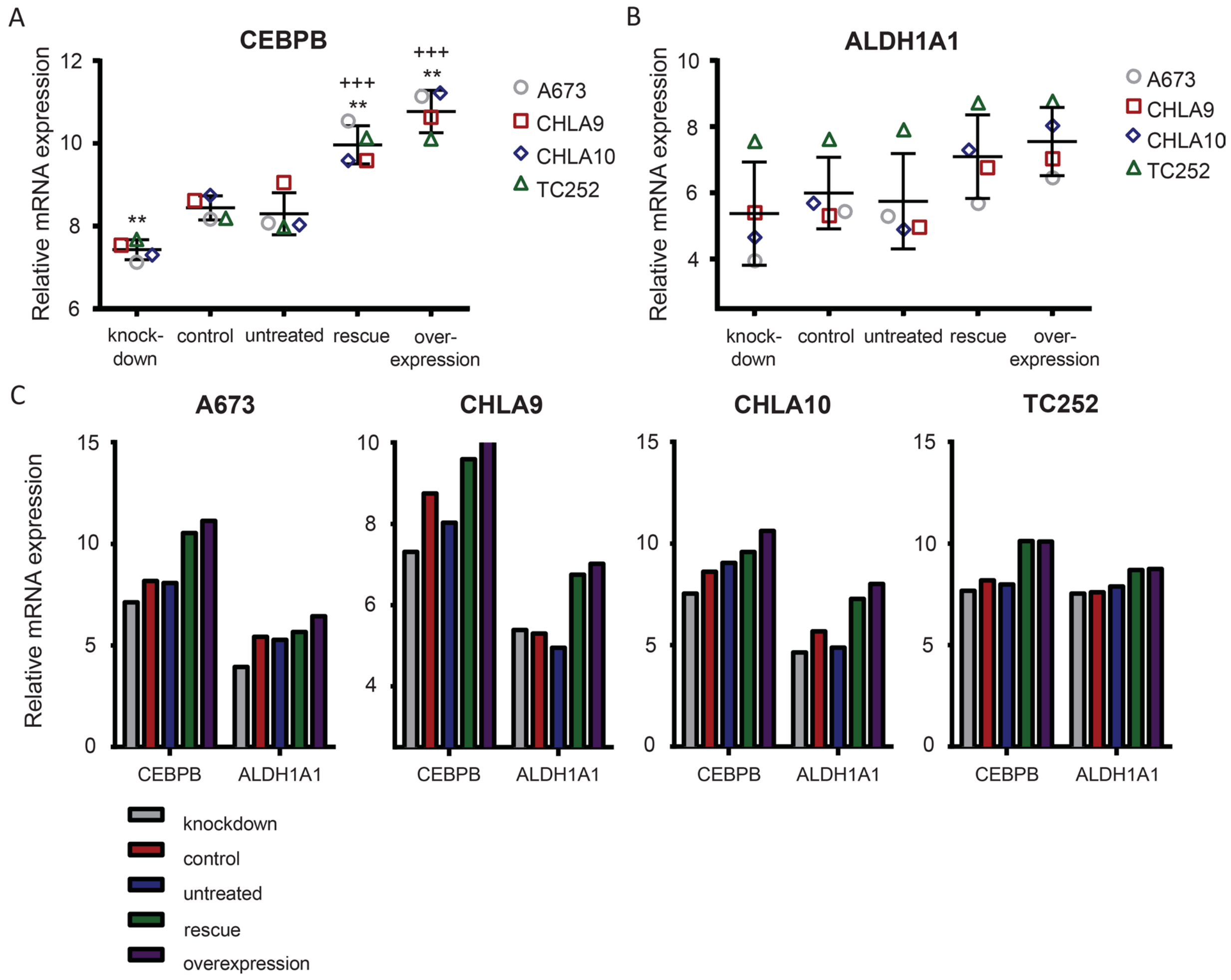

Supplementary Figure 3: Microarray individual gene mRNA expression. *CEBPB* (**A**) and *ALDH1A1* (**B**) probe levels in Ewing sarcoma cell lines run on the Human Transcriptome Array 2.0 (Affymetrix). Each cell line underwent C/EBPβ knockdown, control treatment, no treatment, C/EBPβ-1 rescue, and C/EBPβ-1 overexpression. Multiple t tests were used to determine statistical significance compared to control, using cell lines as replicates for each treatment. (*P*-value for experimental vs control: $^{*}P < 0.05$, $^{**}P < 0.005$, $^{***}P < 0.0001$; *P*-value for experimental vs knockdown: $^{+}P < 0.05$, $^{++}P < 0.005$, $^{+++}P < 0.0001$). (**C**) Microarray mRNA expression levels of *CEBPB* and *ALDH1A1* in each cell line individually.

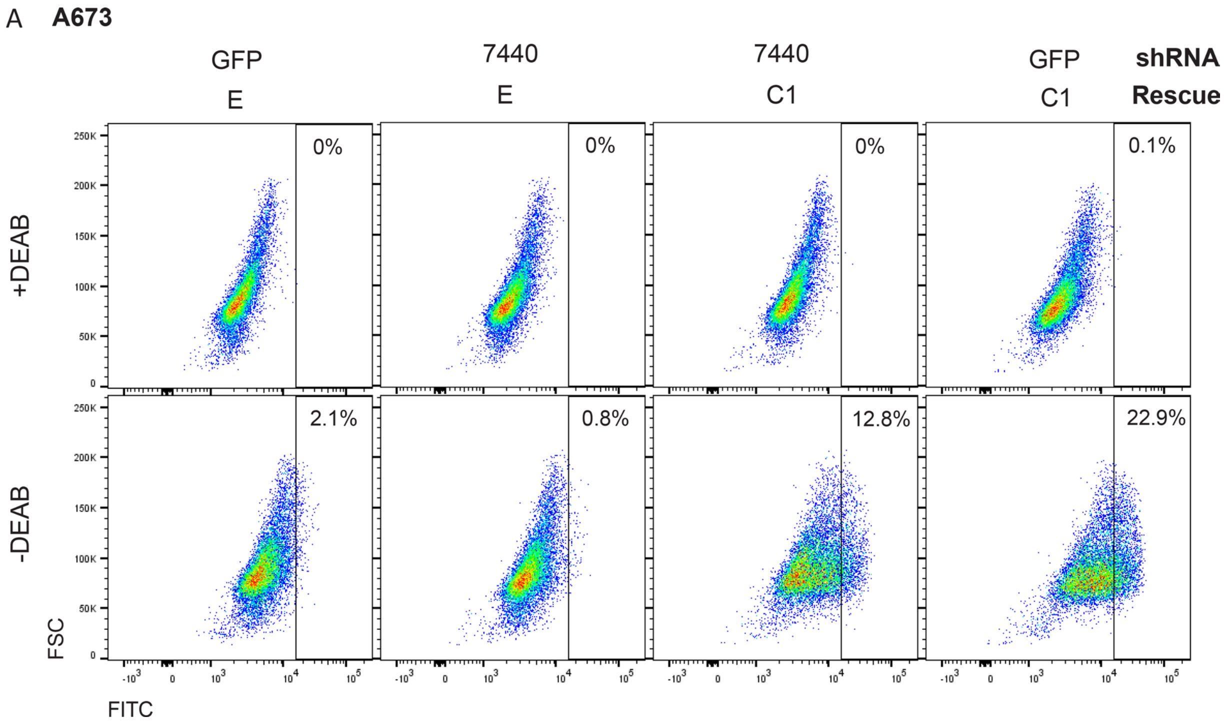

Supplementary Figure 4: ALDH activity is regulated by C/EBPβ expression. (**A**) Representative FACS analysis of A673 cells subject to the Aldefluor Assay from Figure 6C. Percentage of viable cells that are gated as ALDH-high is shown.

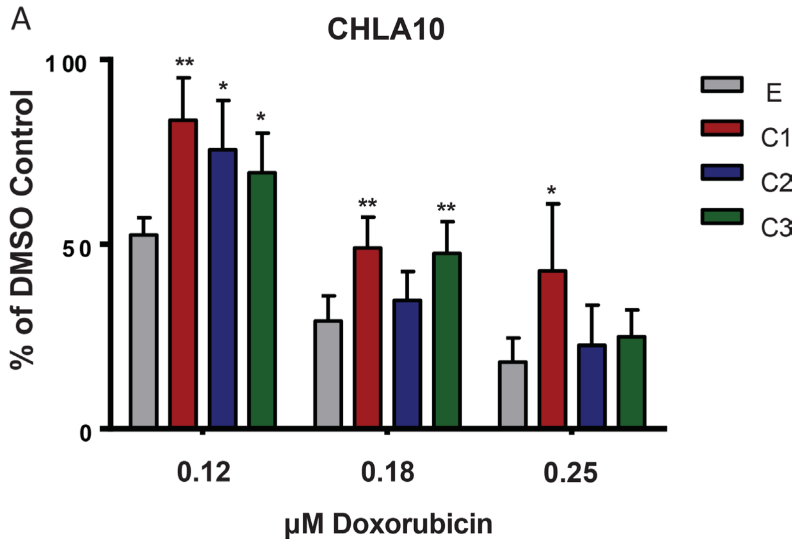

Supplementary Figure 5: C/EBPβ overexpression leads to chemoresistance in Ewing sarcoma cells. (**A**) Each of the C/EBPβ isoforms were overexpressed in CHLA10 cells. Cells were treated with the indicated concentrations of doxorubicin for 48 hours and viability was measured. Multiple t tests were performed to statistically compare isoform overexpression with control. (*P*-value for experimental vs control: $*P < 0.05$, $**P < 0.005$, $***P < 0.0001$).

Supplementary Table 1: Most differentially expressed genes between C/EBPβ-1 knockdown and overexpression

Gene Symbol	Average fold change-overexpression to knockdown	Average fold change-knockdown to control	Average fold change-overexpression to control	*P*-value (*t*-test)
HSD11B1	13.25738198	1.323867054	16.79922764	0.0122
PI3	10.29593539	2.452617067	7.977039011	0.0157
SQRDL	4.786275694	0.967203084	2.657827857	0.0086
ALDH1A1	4.525504871	1.002723786	3.333645432	0.0047
HCAR2	4.281326169	1.708332145	3.273052326	0.0144
S100A8	3.673264414	1.591140243	3.837087507	0.0004
CHI3L1	3.649848927	1.330458952	4.395477804	0.0180
BEX1	3.302117026	1.113400588	3.458843328	0.0052
MAB21L3	3.046550701	1.053816373	2.165147033	0.0009
ARG1	2.775766573	1.099020845	3.235024564	0.0205
ELOVL3	2.743594465	1.188537975	3.108500189	0.0164
CAMK1G	2.681975392	0.71105805	1.515971187	0.0013
TMEM71	2.440919133	1.104308684	2.457258554	0.0032
TDRD9	2.381657982	1.108036956	2.675158428	0.0124
PYGL	2.22111024	1.100531356	1.749663719	0.0026
LINC00277	1.789361028	0.971203987	1.30691683	0.0030
SLC36A4	1.696229641	0.806542801	1.439591012	0.0098
HEBP2	1.667031944	1.047796465	1.346806572	0.0039
GNG4	1.663858792	0.765941174	1.228887061	0.0058
ALDH2	1.6633151	0.951264866	1.346590632	0.0037
MCUR1	1.629905985	0.739833355	1.17041027	0.0028
RAP2A	1.607884471	0.903839179	1.294526352	0.0062
ADAMTS5	1.589341061	0.952812833	1.48134225	0.0031
TMX4	1.576035738	0.918479228	1.380549866	0.0038
CBR4	1.523126482	0.762719505	1.155010526	0.0051
NDRG1	1.514096072	1.163549228	1.550151979	0.0020
FZD6	1.493979136	0.782822024	1.202877172	0.0063
TMEM170A	1.487303939	0.852365142	1.154014219	0.0007
CD1D	1.484965074	1.087098751	1.407233111	0.0091
RNF122	1.483481823	0.927656116	1.187463361	0.0050
ACAP2	1.449646863	1.007071769	1.474510402	0.0099
KLHL11	1.445858631	0.762226145	0.991423023	0.0049
CNIH4	1.43888022	0.810645046	1.210895752	0.0035
H6PD	1.423634944	1.063354764	1.365710787	0.0023
EIF5A2	1.418139544	0.86657814	1.114242049	0.0052
MPP1	1.358302444	1.012508135	1.238091838	0.0067
EIF4A1P2	1.349750499	0.934561007	1.180348598	0.0054
TGFBR3	1.338296935	0.99740753	1.244532581	0.0014
HEBP1	1.32764506	1.00159794	1.250025333	0.0019
B3GNT2	1.325583913	0.882428169	1.154059814	0.0031
HIST1H2BF	1.299831846	0.917522537	1.149959724	0.0037
ZNF826P	1.292644443	0.94025534	1.169411216	0.0051
PTMAP5	1.279311199	0.904440656	1.129039009	0.0049
